# Phase I trial and pharmacokinetic study of berubicin (RTA 744) in patients with recurrent or refractory high-grade gliomas

**DOI:** 10.1093/noajnl/vdag150

**Published:** 2026-07-31

**Authors:** Charles A Conrad, Sigmund H Hsu, Neil Thapar, Elizabeth A Maher, Timothy F Cloughesy, Howard Colman, Mark R Gilbert, Vinay K Puduvalli, Timothy Madden, Waldemar Priebe

**Affiliations:** Department of Neuro-Oncology, University of Texas M.D. Anderson Cancer Center, Houston, Texas, USA; US Oncology, The Woodlands, Texas, USA (C.A.C., M.R. G.); Department of Neuro-Oncology, University of Texas M.D. Anderson Cancer Center, Houston, Texas, USA; Department of Blood and Marrow Transplantation, The University of Texas M.D. Anderson Cancer Center, Houston, Texas, USA; Reata Pharmaceutical Inc., Dallas, Texas, USA; Center for Neuro-Oncology, Dana-Farber Cancer Institute, Boston, Massachusetts, USA; Department of Medical Oncology, Dana-Farber Cancer Institute and Harvard Medical School, Boston, Massachusetts, USA; Department of Neurology and Henry E. Singleton Brain Tumor Program, David Geffen School of Medicine, University of California Los Angeles, Los Angeles, California, USA; Department of Neuro-Oncology, University of Texas M.D. Anderson Cancer Center, Houston, Texas, USA; Department of Neuro-Oncology, University of Texas M.D. Anderson Cancer Center, Houston, Texas, USA; National Cancer Institute, Bethesda, Maryland, USA; US Oncology, The Woodlands, Texas, USA (C.A.C., M.R. G.); Department of Neuro-Oncology, University of Texas M.D. Anderson Cancer Center, Houston, Texas, USA; Department of Experimental Therapeutics, University of Texas M.D. Anderson Cancer Center, Houston, Texas, USA; Department of Experimental Therapeutics, University of Texas M.D. Anderson Cancer Center, Houston, Texas, USA

**Keywords:** anthracycline, berubicin, blood–brain barrier, glioblastoma, phase I clinical trial

## Abstract

**Background:**

This multi-institutional phase I trial aimed to determine the maximum tolerated dose (MTD), dose-limiting toxicities, pharmacokinetics, and preliminary antitumor activity of berubicin (WP744 or RTA744), designed to cross the blood–brain barrier, in patients with primary brain cancers.

**Methods:**

Thirty-five patients with recurrent or refractory primary brain cancers, including glioblastoma multiforme, received berubicin infusions over 2 h for 3 consecutive days (one course) every 21 days. Daily doses escalated from 1.2 to 9.6 mg/m^2^ using an accelerated titration design. Plasma levels of berubicin were measured via high-performance liquid chromatography–tandem mass spectrometry to estimate pharmacokinetic parameters.

**Results:**

The daily MTD was determined to be 7.5 mg/m^2^. Nonhematological toxicities were minimal; no cardiotoxicity was observed. In the intention-to-treat population (*n* = 35), 1 patient had a durable complete response, 1 had a partial response, and 9 had stable disease, corresponding to an objective response rate of 5.7% and a disease control rate of 31.4%. In the response-evaluable subset (*n* = 25), the corresponding rates were 8% and 44%, respectively; these secondary estimates should be interpreted with caution as patients who discontinued early were excluded. Pharmacokinetic analysis determined a mean half-life of 32.8 h. The area under the curve increased proportionally with dose.

**Conclusions:**

The tolerability and efficacy of berubicin, including one durable complete response, warrant continued development of the molecule. Berubicin shows activity over a range of doses, including a durable response at 2.4 mg/m^2^. The recommended phase II dose is 7.5 mg/m^2^ infusions over 2 h for 3 days every 3 weeks.

**Trial registration:**

NCT00526812.

Key Points

- Berubicin was safe at a daily maximum tolerated dose of 7.5 mg/m^2^; neutropenia was the main dose-limiting toxicity.
- In the intention-to-treat population, a 31.4% disease control rate was observed, with one complete response and one partial response (a 5.7% response rate).
- Activity was seen across multiple doses, including a durable response at 2.4 mg/m^2^.

Importance of the Study

This study highlights the potential of berubicin in patients with primary brain cancers, including glioblastoma multiforme. Due to its lipophilic properties, berubicin has been shown in preclinical studies to penetrate the blood–brain barrier, addressing a critical challenge in treating malignant gliomas. In the intention-to-treat population, berubicin achieved a 31.4% disease control rate and an observed response rate of 5.7% with minimal nonhematological toxicity. The complete response observed supports further study, although its biological significance is uncertain given the absence of molecular characterization. These findings support further investigation of berubicin in phase II trials at the daily maximum tolerated dose of 7.5 mg/m^2^ and exploration of its use in combination regimens or adjuvant settings. Additionally, the study adds to the fairly limited literature on effective chemotherapeutic agents for brain tumors and supports continued clinical investigation in high-grade gliomas.

In the United States, the estimated number of new cases of brain and other nervous system cancer for 2024 is 25 400, with 18 760 related deaths expected.^1^ Glioblastoma multiforme (GBM) is the most common malignant primary brain tumor in adults and remains associated with very poor long-term survival. Highly invasive and virtually incurable, GBM has a 5-year overall survival rate of less than 7%.^2^ For several decades, the survival outcomes for patients with GBM have changed very little, with a median survival time of 14-16 months.^3^

For context, it is important to share that this study was conducted from November 7, 2005 (first patient enrolled) through November 14, 2008 (last patient completed). As this was before routine integration of molecular markers into glioma classification, histologies are described using terminology current at the time of the study, with WHO 2021 terminology provided where feasible.

The standard of care for newly diagnosed glioblastoma consists of maximal safe resection followed by concurrent radiation therapy with temozolomide, followed by adjuvant temozolomide.^4^^,^^5^ This treatment typically yields an increase in median survival from 12.1 to 14.6 months and an increase in 2-year overall survival to 27.2% (vs 10.9% with radiotherapy alone).^4^ More recently, the addition of tumor-treating fields (TTFields) to maintenance temozolomide further improved outcomes.^6^

Despite advances in molecularly-defined subsets, outcomes remain poor, particularly in recurrent glioblastoma.^7^^,^^8^ There is no established standard of care for patients with recurrent disease and the median overall survival is less than 1 year. Although multiple systemic and immune-based strategies are under investigation, durable responses remain uncommon.^9^

The lack of permeability of the blood–brain barrier to most therapeutics limits treatment of this type of tumor. Temozolomide, a small lipophilic alkylating agent, crosses this barrier effectively, rendering it a viable chemotherapeutic option for brain tumors. However, the continued development of small molecules capable of crossing the blood–brain barrier, which evade efflux by active transport, is of critical importance.

Berubicin (WP744 or RTA 744) was designed from the conserved doxorubicin molecular structure and tailor-made for improved lipophilicity, while preserving DNA binding and acting as a potent topoisomerase II poison. Structurally, the 4′-O-benzyl substituent was covalently bound to the glycone ring of the anthracycline. Berubicin was selected from the screening of a series of modified anthracycline analogs as a lead compound for its ability to circumvent ATP-binding cassette transporters Multidrug Resistant Protein-1 (MRP1), P-glycoprotein (P-gp), and its ability to penetrate and cross the blood–brain barrier in preclinical studies,^7^ differentiating it from other clinically available anthracyclines. The mechanism(s) of the cytotoxicity of berubicin seems to be similar to that of doxorubicin, and it is believed to be a topoisomerase II poison. With 50% inhibitory concentration values in the mid-nanomolar range, berubicin was observed to be a more potent topoisomerase II poison than doxorubicin in vitro.^8^ In preclinical trials, the molecule was shown to be active against multidrug-resistant tumors and able to overcome efflux transporters.^8^^,^^9^ Berubicin has also been explored clinically outside primary GBM, including in studies of CNS-involved metastatic disease and leptomeningeal disease (ClinicalTrials.gov: NCT00538343, NCT00512460).

The strong preclinical data for berubicin in the setting of primary brain malignancies were sufficient to prompt this first-in-human, phase I trial (ClinicalTrials.gov: NCT00526812). To balance efficacy and safety while managing the dose-limiting toxicity (DLT) of myelosuppression, typical of anthracycline therapy, berubicin is administered via short daily intravenous infusion over 3 consecutive days every 3 weeks. Of note, this dosing schedule matched the most common schedule of anthracycline dosing at the time the study was performed.^10^

The primary objectives of this study were to determine the DLTs and maximum tolerated dose (MTD) of berubicin administered as a 2-h intravenous infusion for 3 consecutive days every 3 weeks to patients with recurrent or refractory glioblastoma, astrocytoma, or other primary brain cancer. The secondary objectives were to characterize the multiple-dose pharmacokinetics of berubicin, document any potential antitumor activity in those patients with measurable disease, and correlate pharmacokinetic (PK) information with clinical responses of safety and efficacy, aiming to define the recommended dose for future studies.

## Methods

Of note, this study was conducted prior to WHO 2021 CNS tumor classification. Diagnoses were assigned per institutional pathology criteria current at the time (∼2005-2008), and molecular markers (eg IDH, 1p/19q) were not systematically obtained.

### Patient Selection and Eligibility Criteria

Patients were enrolled in this phase I trial (NCT00526812) from 3 institutions in the United States: The University of Texas MD Anderson Cancer Center, The University of Texas Southwestern Medical Center in Dallas, and the University of California, Los Angeles, UCLA Medical School. Eligible patients were adults aged ≥18 years with histologically confirmed recurrent or refractory primary brain cancers for which there were no known effective therapies. There was no limit to the number of prior treatments or extent of surgical resections, provided patients had a Karnofsky Performance Status (KPS) ≥60% and a projected life expectancy of >12 weeks. Patients were required to have a prior histologic diagnosis of an anaplastic astrocytoma (AA), anaplastic oligodendroglioma (AO), anaplastic mixed oligoastrocytoma, GBM, or gliosarcoma (GS), based on tumor tissue obtained at initial diagnosis and/or a prior surgical procedure. Histologic confirmation was required for study eligibility, but repeat biopsy at the time of recurrence/progression was not mandated. Unequivocal evidence of recurrence or progression via neuroimaging procedure was required. Patients diagnosed with lower-grade gliomas were eligible if there was histological evidence that the tumor had progressed to a higher-grade malignancy as specified above.

Patients were required to have adequate hematologic and organ function, defined as hemoglobin ≥9 g/dL, absolute neutrophil count ≥1500/μL, platelets ≥100 000/μL, hepatic enzymes (aspartate aminotransferase [AST] and alanine aminotransferase [ALT]) ≤3× the upper limit of normal (ULN), total bilirubin ≤1.5× ULN, serum creatinine ≤1.5× ULN, and 24-h creatinine clearance (CL) ≥50 mL/min. Any surgical resection prior to enrollment must have been performed at least 2 weeks before study entry, and patients must have fully recovered from any side effects of such therapy. Patients were required to be on a stable dose of corticosteroids for at least 7 days prior to magnetic resonance imaging (MRI) assessments and could not have any contraindication to MRI.

Exclusion criteria included significant hematopoietic, hepatic, or renal dysfunction or uncontrolled medical conditions such as diabetes, active infection, HIV infection, hypertension, congestive heart failure, arrhythmias, or impaired cardiac function, as indicated by any of the following: left ventricular ejection fraction <45% (as determined by multigated acquisition [MUGA] scan or echocardiogram), complete left bundle branch block, obligate use of a cardiac pacemaker, congenital long QT syndrome, significant bradycardia, QT_c_ >480 ms, angina pectoris, or myocardial infarction within 6 months prior to starting the study drug. Patients receiving medications known to cause QT interval prolongation were also excluded. Additional exclusion criteria included therapeutic doses of warfarin, 24-h total urinary protein >500 mg, or receipt of the following types of prior or concurrent therapy (or failure to recover from the toxic effects of such therapy prior to study entry): investigational drugs within 4 weeks; chemotherapy within 4 weeks (6 weeks for any nitrosourea or mitomycin-C, 2 weeks for vincristine); metronomic daily dosing of chemotherapy agents (patients could be enrolled 1 week after discontinuation of these agents if the absolute neutrophil count had returned to ≥1000/μL and platelets to ≥100 000/μL); radiation therapy within 2 weeks; and biologic therapy, immunotherapy, or cytostatic agent therapy within 4 weeks. Patients with previous implantation of polifeprosan–carmustine wafers (Gliadel Wafer; Eisai Corporation, Woodcliff Lake, NJ) were eligible if there was evidence of disease progression and if the wafer was implanted at least 6 weeks prior to study entry.

Additional exclusion criteria were individuals who were pregnant or breastfeeding patients and those not using effective birth control, patients who had any surgery other than resection of a brain tumor within 2 weeks prior to study entry, and patients unwilling or unable to comply with the protocol.

### Study Design

This was a phase I, multicenter, open-label, dose-escalation study of berubicin administered intravenously over 2 h daily for 3 consecutive days, repeated every 3 weeks (1 cycle). The starting dose of berubicin was 1.2 mg/m^2^ daily. Initial dose escalations proceeded according to an accelerated titration design, with each patient treated at twice the dose of the previous patient until any grade 2 or greater toxicity occurred. In the presence of grade 2 or 3 treatment-related toxicity in the first cycle (unrelated to disease progression, intercurrent illness, or concomitant medication), further dose escalations were performed according to a modified Fibonacci scheme, with cohorts expanded to 3 patients. If 2 of 6 patients in any dose cohort experienced a DLT, the previous dose level was declared the MTD. A minimum of 6 patients had to be treated at the MTD to confirm its status. If only 1 of 6 patients experienced a DLT, dose escalation could continue. Intra-patient dose escalation was not allowed.

Toxicities were graded according to the National Cancer Institute Common Terminology Criteria for Adverse Events (CTCAE) version 3.0.^11^ A DLT was defined as any grade 4 hematologic toxicity; grade ≥3 neutropenic fever or nonhematologic toxicity (excluding nausea, vomiting, or diarrhea that could be controlled with prophylactic therapy); grade ≥2 proteinuria, hematuria, or neurotoxicity; or any serum creatinine or total bilirubin value >2× ULN. Grade 3 elevations of AST/ALT were considered DLT only if persistent for >7 days. Other toxicities considered to be DLTs were delays of more than 1 day in administering the second or third daily dose due to toxicity expected to lead to a DLT, or delays in starting the next cycle of treatment by more than 7 days to allow for recovery from toxicity occurring during the first cycle.

Berubicin was formulated and vialed by the University of Iowa Pharmaceutical Services Division, College of Pharmacy. It was provided by the study sponsor, Reata Pharmaceuticals, Inc. (Dallas, TX), as an aqueous solution in sterile 5 mL or 30 mL vials containing 1 mg/mL of active pharmaceutical ingredient in sterile water for injection, adjusted to pH 3 with hydrochloric acid. The final solution of berubicin was admixed in 250 mL of 10% dextrose in water and infused over 2 h at a rate of 125 mL/h via a patient’s central line.

### Safety and Tolerability Assessments

At the start of each cycle, patients underwent a history and physical examination; assessment of performance status; coagulation studies, including fibrinogen and prothrombin/partial thromboplastin time; complete blood count with differential; measurement of AST, ALT, total bilirubin, alkaline phosphatase, serum electrolytes, blood urea nitrogen, and creatinine; urinalysis; and documentation of measurable or assessable disease by physical examination and MRI. Adverse events were recorded throughout the study and graded according to CTCAE version 3.0.^11^ Due to the potential for cardiotoxicity, a class effect of anthracycline-based chemotherapies, routine 12-lead electrocardiograms, and MUGA scans or echocardiograms were performed after each cycle.

Efficacy analyses were performed on patients who received at least 2 cycles of treatment. Tumor restaging was performed every 2 cycles, and antitumor activity was assessed by radiologic image review based on Macdonald criteria.^12^ Time-to-event endpoints such as progression-free survival and overall survival were not prospectively or systematically collected as prespecified efficacy endpoints in this phase I study.

### PK Analysis

The pharmacokinetics of berubicin were evaluated in plasma and urine during the first treatment cycle. The PK analysis set included all patients who took at least one dose, had no major protocol deviations/violations, and had at least one quantifiable plasma or urine concentration collected postdose. Blood samples were obtained from a peripheral vein contralateral to the infusion site on days 1-3 (during dosing) and at 24 and 48 h after the final dose, respectively. On day 1, blood samples were collected at predose, 0.25, 0.5, 1, 2 (end of infusion), 4, 6, 8, 12, and 24 h after the start of infusion. On days 2 and 3, samples were collected at 2, 4, 6, 8, 12, and 24 h after infusion start. A final blood sample was collected 48 h after the start of the third infusion. Plasma was isolated within 30 min of collection, centrifuged at 800 × *g* for 15 min at 4 °C, and stored at −70 °C until analysis. Urine was collected over 24 h on day 1.

Plasma and urine concentrations of berubicin were measured using a validated liquid chromatography–tandem mass spectrometry method developed at The University of Texas MD Anderson Cancer Center. Plasma concentration–time data were analyzed using noncompartmental methods with WinNonLin version 5.0 (Pharsight Corporation, Mountain View, CA). Pharmacokinetic parameters, including maximum plasma concentration (C_max_), area under the concen­tration–time curve (AUC), CL, volume of distribution, and half-life (t_1/2_), were calculated.

### Statistical Analysis

All statistical analyses of PK data were performed using Phoenix WinNonlin version 8.2 (Certara USA, Inc., Princeton, NJ) and SAS version 9.1.3 (SAS Institute Inc., Cary, NC). Pharmacokinetic parameters were listed for each patient and summarized by dose level using descriptive statistics. Summary statistics included the number of observations (*n*), arithmetic mean, SD, coefficient of variation (CV%), minimum, median, and maximum values. Geometric mean and geometric CV% were calculated for all PK parameters except for time to maximum concentration (t_max_).

Individual plasma concentration–time profiles were plotted for each dose level, and mean concentration–time ­profiles were presented as arithmetic mean ± SE.

### Assessment of Dose Linearity

Dose linearity of berubicin pharmacokinetics was evaluated using a power model. The relationship between dose and PK parameters—specifically, the C_max_ after the third dose (C_max_ of the third dose) and the area under the plasma concentration–time curve from time zero to infinity (AUC_0-∞_)—was assessed.

The linearized power model employed was:


ln(Y) = α+β × ln(dose)

where:

- *Y* = the PK parameter of interest (C_max_ of the third dose or AUC_0-∞_)
- α = the intercept (a constant)
- β = slope (indicator of dose proportionality)

Linear regression analyses were performed on the log-transformed data using the Proc REG procedure in SAS version 9.1.3. A dose-proportional response was considered when the slope (β) was close to 1.00, and the 95% CI for β included 1.00.

### Data Sets Analyzed

All patients who were enrolled and treated were included in the safety population. The efficacy population was those patients who had received at least 2 cycles of treatment, had measurable disease at baseline, and for whom pre- and posttreatment MRIs were available. The MTD population included patients who either experienced a DLT during cycle 1 or completed the full 3-day dosing regimen and at least 18 days of follow-up with all scheduled safety assessments.

## Results

### Patient Characteristics and Treatments

The first patient was enrolled on November 7, 2005; the last patient completed on November 14, 2008. Patient characteristics and prior treatment information are summarized in Table 1. Thirty-five patients (median age 50 years; range 29-70 years) were enrolled across 5 dose levels of berubicin (1.2-9.6 mg/m^2^). The majority were men (69%). The KPS of patients ranged from 60 to 100, with a mean of 80. Histologically, 80% (*n* = 28) had GBM, 11% (*n* = 4) had AO, 6% (*n* = 2) had AA, and 3% (*n* = 1) had GS.

**Table 1.** Baseline patient characteristics (*N* = 35)

Characteristic	Value
Age, years	
Median	50
Range	29-70
Sex	
Male	24
Female	11
Performance status (KPS)	
Median	80
Range	60-100
Histology, no. of patients (%)	
Glioblastoma multiforme	28 (80%)
Gliosarcoma	1 (3%)
Anaplastic oligodendroglioma	4 (11%)
Anaplastic astrocytoma	2 (6%)
Prior surgery for brain tumor	
Yes	35 (100%)
No	0
Type of surgery	
Biopsy only	12 (34%)
Subtotal resection	15 (43%)
Gross total resection	26 (74%)
Prior chemotherapy	
Yes	35 (100%)
No	0
Prior radiation	
Yes	35 (100%)
No	0

Abbreviations: KPS, Karnofsky Performance Status.

All patients had undergone one or more surgeries for brain tumors, most frequently gross total resection (*n* = 26; 74%) followed by subtotal resection (*n* = 15; 43%) and biopsy only (*n* = 12; 34%). Additionally, all patients had previously undergone chemotherapy and radiation therapy.

Eight patients (23%) discontinued the study early (3 patients because of clinical deterioration and 5 others because of progressive disease [PD]) prior to initiating a second course of therapy and were not eligible for efficacy evaluation. An additional 2 patients (5.7%) were not included in the efficacy population as they did not have measurable disease at baseline, leaving 25 of the 35 enrolled patients evaluable for radiographic response.

### Safety and Tolerability

A total of 33 of the 35 enrolled patients met all requirements to be included in the dose-escalation study, the results of which are summarized in Table 2. At the initial dose level of 1.2 mg/m^2^, 1 patient was treated without incident. At 2.4 mg/m^2^, 3 patients were enrolled; there were no CTCAE grade 2 or higher DLTs. Dose escalation proceeded to 4.8 mg/m^2^, where one patient was treated without any CTCAE grade 2 or higher DLTs. Dosing was escalated to 9.6 mg/m^2^.

**Table 2.** Dose-limiting toxicities observed determining maximum tolerated dose

Daily dose (mg/m²)	No. treated (*n*)	No. with ≥1 DLT (*n*, %)	**DLTs observed** ^a^	Action taken
1.2	1	0 (0 %)	–	Escalate
2.4	3	0 (0 %)	–	Escalate
4.8	1	0 (0 %)	–	Escalate
9.6	7	3 (43 %)	Neutropenia (*n* = 2), thrombocytopenia (grade 4); hydrocephalus, fatigue, muscle wasting (grade 3)	De-escalate to 7.5
7.5	20	2 (10 %)	Hypokalemia, fatigue (grade 3)	Declared MTD

a Grades per CTCAE v3.0; only first-cycle events were counted as DLTs. At the 9.6 mg/m^2^ dose, 3 of 7 patients experienced ≥1 DLT: 2 with life-threatening neutropenia—1 of whom also had thrombocytopenia, fatigue and muscle wasting—and 1 with grade 3 hydrocephalus.

Abbreviations: CTCAE, Common Terminology Criteria for Adverse Events; DLT, dose-limiting toxicity; MTD, maximum tolerated dose.

During cycle 1, at the 9.6 mg/m^2^ dose level, significant toxicities emerged among the 7 patients receiving treatment; DLTs were reported in 3 patients (43%)—neutropenia alone in one patient, hydrocephalus in one, and fatigue, muscle wasting, and thrombocytopenia in another patient with neutropenia. Per protocol, the dose was reduced to 7.5 mg/m^2^, and 20 patients were treated at this dose. As only 2 (10%) patients experienced DLTs at this reduced dose (hypokalemia and fatigue), the MTD was determined to be 7.5 mg/m^2^.

Non-DLT treatment-emergent adverse events were predominantly grade 1-2 and were managed with routine supportive care. The most frequent events (≥10% of patients) were gastrointestinal or systemic in nature, led by fatigue (42.6%), headache (40.7%), nausea/vomiting (27.8%), and constipation (24.1%). Hematologic laboratory abnormalities—primarily transient lymphopenia (53.7%) and leukopenia (37.0%)—were also common. Neurologic manifestations consistent with underlying disease (pyramidal-tract signs, gait disturbance, and speech disorder) occurred in roughly one-third of patients but did not require dose modification. Serial MUGA and echocardiography assessments showed no clinically meaningful declines in left-ventricular ejection fraction. QT-corrected intervals remained stable, with no new-onset arrhythmias, supporting the absence of anthracycline-associated cardiotoxicity in this cohort.

### PK Evaluation

Pharmacokinetic sampling was conducted during the first treatment cycle, with samples collected from the start of the daily intravenous infusion of berubicin up to 48 h after the initiation of the final dose. Pharmacokinetic parameters for the full sampling regimen are summarized in Table 3 and for each dose in Table 4. At the 2 highest doses—7.5 mg/m^2^ and 9.6 mg/m^2^—the C_max_ observed at the end of the first day of administration were 16.3 and 14.9 ng/mL, respectively.

**Table 3.** Summary of berubicin pharmacokinetic parameters over first dosing regimen

	Geometric mean parameters (CV%)							
Dose (mg/m²)	C_ss_ (ng/mL)	AUC_0-last_ (h × ng/mL)	AUC_0-∞_ (h × ng/mL)	t_1/2_ (h)	V_z_ (L/m²)	CL (L/h/m²)	UE (μg/m² [24 h])	UE% (%)
1.2 (*n* = 1)	0.764 (NA)	68.5985 (NA)	88.027 (NA)	32.0639 (NA)	1891.807 (NA)	40.8965 (NA)	46.400 (NA)	2.100 (NA)
2.4 (*n* = 3)	1.729 (23.8%)	150.7367 (24.1%)	193.8654 (26.7%)	34.8587 (12.5%)	1867.745 (24.0%)	37.1392 (26.7%)	88.457 (56.2%)	1.867 (48.9%)
4.8 (*n* = 2)	2.972 (18.3%)	261.4908 (9.3%)	323.3781 (5.8%)	25.4374 (45.8%)	1634.179 (39.2%)	44.5299 (5.8%)	124.069 (49.3%)	1.421 (51.3%)
7.5 (*n* = 20)	5.461 (43.9%)	445.1048 (45.1%)	539.0903 (45.7%)	27.2251 (33.0%)	1639.326 (53.7%)	41.737 (45.7%)	596.926 (83.5%)	4.002 (88.8%)
9.6 (*n* = 7)	5.97 (49.2%)	499.6604 (43.5%)	663.1073 (43.9%)	36.3207 (50.3%)	2275.82 (56.8%)	43.4319 (43.9%)	255.500 (82.8%)	1.360 (68.5%)

Values are presented as geometric means (geometric CV%).

Abbreviations: AUC_0-last_: area under the plasma concentration–time curve from time 0 to time of the last quantifiable concentration calculated by the linear up/log linear down trapezoidal method; AUC_0-∞_: area under the plasma concentration–time curve from time 0 to infinite time, calculated as the sum of AUC_0-last_ and C_last_/λ_z_, where C_last_ is the last observed quantifiable concentration; CL: total body clearance; C_ss_: average concentration, calculated as the geometric mean of concentrations over the 72-h dosing interval; CV%, coefficient of variance; NA, not applicable; t_1/2_: elimination half-life associated with the terminal slope (λ_z_) of the log-linear drug concentration–time curve, calculated as ln(2)/λ_z_; UE: The amount of urinary excretion of berubicin drug in the first 24 h post dose; UE% The percent of urinary excretion of berubicin drug in the first 24 h post dose; V_z_: volume of distribution.

**Table 4.** Detailed PK parameters of berubicin for each dose

	First dose			Second dose			Third dose			
Dose (mg/m²)	t_max_ (h)	C_max_ (ng/mL)	AUC_0-tau_ (h × ng/mL)	t_max_ (h)	C_max_ (ng/mL)	AUC_0-tau_ (h × ng/mL)	t_max_ (h)	C_max_ (ng/mL)	AUC_0-tau_ (h × ng/mL)	Rac
1.2 (*n* = 1)	2.067 (2.067-2.067)	1.500 (NA)	12.2412 (NA)	2.000 (2.000-2.000)	1.390 (NA)	17.8600 (NA)	2.000 (2.000-2.000)	1.700 (NA)	23.3600 (NA)	1.9083 (NA)
2.4 (*n* = 3)	2.000 (1.000-2.033)	3.740 (40.9%)	26.4908 (21.6%)	2.000 (2.000-2.000)	3.651 (8.8%)	37.9113 (15.0%)	2.000 (2.000-2.000)	6.428 (28.8%)	53.4409 (32.9%)	2.0173 (17.2%)
4.8 (*n* = 2)	1.475 (1.000-1.950)	5.823 (64.4%)	47.0652 (11.7%)	2.000 (2.000-2.000)	5.916 (32.5%)	62.2197 (4.8%)	3.000 (2.000-4.000)	7.454 (85.2%)	101.7251 (33.4%)	2.1614 (21.0%)
7.5 (*n* = 20)	2.000 (0.250-6.000)	16.333 (61.1%)	94.6672 (44.3%)	2.000 (2.000-23.633)	16.053 (66.6%)	125.8335 (45.0%)	2.000 (0.000-4.000)	17.214 (67.1%)	148.1024 (55.0%)	1.5997 (36.2%)
9.6 (*n* = 7)	2.000 (1.000-2.267)	14.858 (104.0%)	100.6074 (60.3%)	2.017 (2.000-2.217)	15.578 (44.1%)	133.1834 (33.9%)	2.000 (2.000-2.183)	17.685 (72.6%)	163.7801 (45.2%)	1.6279 (38.7%)

All values except t_max_ are presented as geometric means (geometric CV%), t_max_ presented as a median (range).

Abbreviations: AUC_0-tau_: area under the plasma concentration–time curve from time 0 to tau, where tau is the dosing interval, calculated by the linear up/linear down trapezoidal method; C_max_: Maximum plasma concentration during each of the 3 dosing intervals (observed); CV%, coefficient of variance; NA, not applicable; Rac: the accumulation ratio calculated as AUC_0-tau_ (3rd dose)/AUC_0-tau_ (1st dose); t_max_: time from each dose to reach the maximum plasma concentration for each of the 3 dosing intervals (observed).

When data from all doses were combined, the mean terminal t_1/2_ of berubicin was 32.8 h, and mean CL was 41.5 L/h/m^2^. Drug accumulation was evident after 3 consecutive days of infusion, with an accumulation ratio of 1.91 ± 0.21. The AUC for berubicin was proportional to the administered doses (data not shown), indicative of linear pharmacokinetics. Renal elimination of berubicin was minimal, as expected for an anthracycline, averaging 2.2 ± 1.1%.

### Efficacy

**Figure 1.**
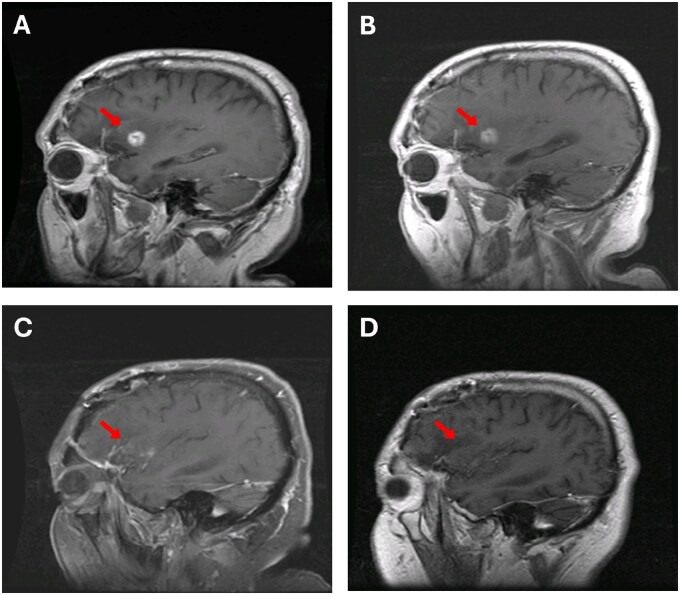
MRI of patient achieving complete response after 7 cycles of berubicin. A 52-year-old man with recurrent GBM that had progressed from AA experienced a complete response after 7 cycles of berubicin at a dose of 2.4 mg/m^2^/day. This CR was confirmed via follow-up MRI. (A) Before berubicin (January 20, 2006). (B) After 2 cycles (March 7, 2006). (C) After 6 cycles (July 11, 2006). (D) After 7 cycles (August 1, 2006). AA, anaplastic astrocytoma; CR, complete response; GBM, glioblastoma multiforme; MRI, magnetic resonance imaging.

Analyzing the more conservative intention-to-treat (ITT) population (*n* = 35), the overall response rate (ORR, defined as the proportion of patients achieving a complete response [CR] or partial response [PR] per Macdonald criteria) was 5.7% (2 out of 35 patients). One patient achieved a CR (Figure 1), and another patient achieved a PR. Nine patients had stable disease (SD). Therefore, in the ITT population, the disease control rate (DCR), representing patients who achieved CR, PR, or SD, was 31.4% (11 out of 35 patients). These tumor responses per Macdonald criteria are summarized in Table 5. While SD may be clinically relevant in recurrent glioblastoma, these findings should be interpreted cautiously in this small, nonrandomized phase I cohort.

**Table 5.** Tumor response per Macdonald criteria

Response	*n* (%)	ITT (*n* = 35)	Evaluable (*n* = 25)
Responders	2	5.7%	8.0%
CR	1	2.9%	4.0%
PR	1	2.9%	4.0%
Non-responders	23	65.7%	92.0%
SD	9	25.7%	36.0%
PD	14	40.0%	56.0%
Non-evaluable (ITT)	10	28.6%	–

Abbreviations: CR, complete response; ITT, intention-to-treat; PD, progressive disease; PR, partial response; SD, stable disease.

Of the 35 patients enrolled in the study, 25 (71.4%) received at least 2 cycles of berubicin, had measurable disease at baseline, and had pre- and post-treatment MRIs available and were evaluable for response according to the Macdonald criteria. The most frequent reason for exclusion from the efficacy population was that patients did not receive at least 2 cycles of study drug (*n* = 8/35; 22.9%). The other 2 participants not included in the efficacy population did not have measurable disease at baseline. Patients not receiving ≥2 cycles were considered nonresponders.

Among response-evaluable patients (*n* = 25/35; 71.4%), the ORR was 8% (2 out of 25 patients). Therefore, in the response-evaluable population, the DCR, representing patients who achieved CR, PR, or SD, was 44% (11 out of 25 patients).

## Discussion

This phase I study suggests that berubicin, administered at the daily MTD of 7.5 mg/m^2^, has preliminary antitumor activity and an acceptable safety profile in patients with recurrent high-grade gliomas. The findings support further evaluation of berubicin in phase II trials to assess its efficacy more comprehensively.

Unlike traditional anthracyclines, which cannot cross the blood–brain barrier, the lipophilic properties of berubicin enable it to penetrate this barrier effectively in preclinical studies. This unique characteristic provided the preclinical rationale for clinical investigation in brain tumors. However, direct CNS drug levels, intratumoral exposure, and pharmacodynamic markers were not measured in this trial, so clinical evidence of CNS target engagement remains indirect. The durable CR observed in one patient is notable—remission of disease at last check (∼19 years as of April 2025)—but its biological significance is uncertain given the diagnostic era and absence of molecular characterization.

The safety profile of berubicin was manageable, with neutropenia, hypokalemia, hydrocephalus, fatigue, muscle wasting, and thrombocytopenia identified as DLTs. Non-hematological toxicities were generally mild and included fatigue, headache, nausea and vomiting, and constipation. The tolerability of berubicin supports the feasibility of administering multiple treatment cycles without significant delays or dose reductions, as evidenced by patients receiving up to 9 cycles.

In the ITT population (*n* = 35), the observed ORR was 5.7%, and the DCR was 31.4%. In the response-evaluable patient subset (*n* = 25), the corresponding rates were 8% and 44%. Note, however, that these secondary estimates are susceptible to selection bias because patients who discontinued early were excluded. Taken together, these findings should be considered preliminary and hypothesis-generating, particularly given the small sample size, histologic heterogeneity, nonrandomized design, and absence of molecular profiling.

The current study supports feasibility and tolerability of dosing intravenously over 2 h daily for 3 consecutive days, repeated every 3 weeks at 7.5 mg/m^2^/day. However, as this trial was not designed to compare schedules, schedule optimization would require prospective evaluation (eg PK/PD-guided designs). Future clinical trials of berubicin are warranted to evaluate potential alternate dosing schedules and to assess its efficacy in combination regimens or as adjuvant therapy. These results support continued clinical evaluation of berubicin in high-grade gliomas.

While the results are encouraging, we acknowledge that they are largely hypothesis-generating at this point; they will need to be further supported with larger, well-designed, randomized clinical studies, including definitive molecular profiling.

Of particular importance is the absence of molecular profiling. As molecular features commonly assessed today (eg IDH mutation status, 1p/19q codeletion) were not systematically assessed as part of this study, the results cannot fully be reclassified according to contemporary WHO criteria. This limits the interpretation of the results and introduces uncertainty when comparing these results with modern studies of molecularly defined gliomas. Therefore, the present findings should be interpreted primarily as evidence of safety, pharmacokinetics, and preliminary antitumor activity within a historically defined high-grade glioma cohort, rather than as estimates directly generalizable to current molecularly stratified glioma subgroups. This limitation is especially relevant to the interpretation of the durable CR, as the possibility of a more biologically favorable tumor subtype cannot be excluded.

We acknowledge the substantial interval between study completion and publication. This delay reflects, in part, changes in development stewardship of berubicin over time and the death of the original first author, which complicated completion of the manuscript.

The development of berubicin is currently concentrated in recurrent GBM (NCT04762069). In abstract-reported preliminary results from this phase II trial, berubicin and lomustine demonstrated similar OS and PFS outcomes.^13^ Note that the trial was not powered to determine noninferiority, and this preliminary analysis did not show statistically significant superiority for berubicin.

### Limitations

- While preclinical studies have shown berubicin effectively crosses the blood–brain barrier (BBB) and is retained in brain and brain tumor tissue, direct confirmation of CNS penetration and target engagement in humans was not obtained in this trial—CSF drug concentrations, intratumoral exposure measurements, and pharmacodynamic biomarkers were not collected.
- As the study was conducted before routine integration of molecular markers into glioma classification, histologies are described using terminology current at the time of the study.
- As overall survival and progression-free survival were not systematically collected, efficacy results do not include time-to-event analysis and are limited to radiographic response-based assessments.
- Response-evaluable efficacy estimates are limited by exclusion of patients who did not complete ≥2 cycles, which may inflate response estimates.
- The observed efficacy should be interpreted cautiously given the relatively small sample size, heterogeneity of tumor types, and nonrandomized design.

## Data Availability

Data generated in this study will be made available upon reasonable request by contacting the corresponding author.
